# Real-life benefit of artificial intelligence-based fracture detection in a pediatric emergency department

**DOI:** 10.1007/s00330-025-11554-9

**Published:** 2025-04-07

**Authors:** Maria Ziegner, Johanna Pape, Martin Lacher, Annika Brandau, Tibor Kelety, Steffi Mayer, Franz Wolfgang Hirsch, Maciej Rosolowski, Daniel Gräfe

**Affiliations:** 1 Department of Pediatric Surgery, University Hospital, Leipzig, Germany; 2 Department of Pediatric Radiology, University Hospital, Leipzig, Germany; 3 Institute for Medical Informatics, Statistics and Epidemiology, Leipzig University, Leipzig, Germany

**Keywords:** Artificial intelligence, Appendicular skeleton, Fracture, Medicolegal, Pediatric

## Abstract

**Objectives:**

This study aimed to evaluate the performance of an artificial intelligence (AI)-based software for fracture detection in pediatric patients within a real-life clinical setting. Specifically, it sought to assess (1) the stand-alone AI performance in real-life cohort and in selected set of medicolegal relevant fractures and (2) its influence on the diagnostic performance of inexperienced emergency room physicians.

**Materials and methods:**

The retrospective study involved 1672 radiographs of children under 18 years, obtained consecutively (real-life cohort) and selective (medicolegal cohort) in a tertiary pediatric emergency department. On these images, the stand-alone performance of a commercially available, deep learning-based software was determined. Additionally, three pediatric residents independently reviewed the radiographs before and after AI assistance, and the impact on their diagnostic accuracy was assessed.

**Results:**

In our cohort (median age 10.9 years, 59% male), the AI demonstrated a sensitivity of 92%, specificity of 83%, and accuracy of 87%. For medicolegally relevant fractures, the AI achieved a sensitivity of 100% for proximal tibia fractures, but only 68% for radial condyle fractures. AI assistance improved the residents’ patient-wise sensitivity from 84 to 87%, specificity from 91 to 92%, and diagnostic accuracy from 88 to 90%. In 2% of cases, the readers, with the assistance of AI, erroneously discarded their correct diagnosis.

**Conclusion:**

The AI exhibited strong stand-alone performance in a pediatric setting and can modestly enhance the diagnostic accuracy of inexperienced physicians. However, the economic implications must be weighed against the potential benefits in patient safety.

**Key Points:**

***Question*** *Does an artificial intelligence-based software for fracture detection influence inexperienced physicians in a real-life pediatric trauma population?*

***Findings*** *Addition of a well-performing artificial intelligence-based software led to a limited increase in diagnostic accuracy of inexperienced human readers.*

***Clinical relevance*** *Diagnosing fractures in children is especially challenging for less experienced physicians. High-performing artificial intelligence-based software as a “second set of eyes,” enhances diagnostic accuracy in a common pediatric emergency room setting.*

**Graphical Abstract:**

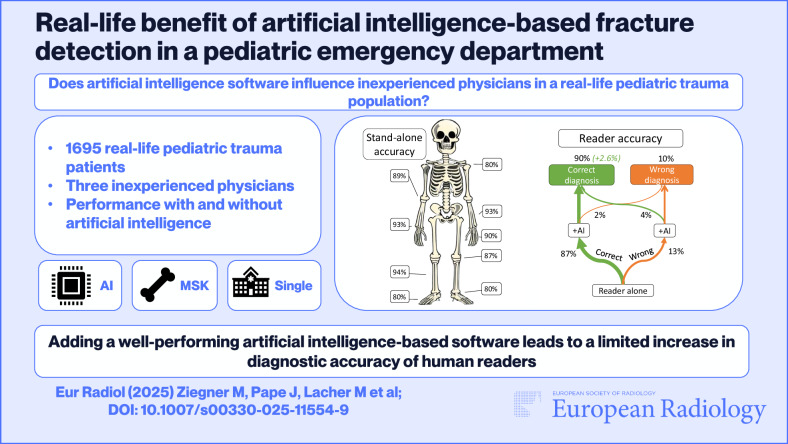

## Introduction

Musculoskeletal injuries are the most common reason for pediatric emergency admissions [1]. Radiographs are often required as part of the diagnostic process to rule out fractures. The initial evaluation of radiographs in the emergency department is frequently conducted ad hoc by non-radiologists, which can result in both false negatives and false positives when compared to the final assessment by a certified pediatric radiologist [2, 3]. Artificial intelligence (AI)-supported software for fracture detection is intended to serve as a “second pair of eyes” and is supposed to improve the performance of frontline physicians and thereby might enhance patient safety and reduce the number of return visits.

Pediatric fractures differ significantly from those of adults, depending on the child’s age and skeletal maturation. The fractures typical or even specific to childhood, bone nuclei that resemble avulsions, and growth plates that mimic fracture lines make the correct diagnosis challenging. The clinical consequences of missed fractures in children are often more severe, potentially resulting in long-term complications such as restricted joint mobility and impaired growth. These outcomes may also carry significant medicolegal implications.

In Europe, 14 deep learning algorithms for fracture assessment in radiographs in adults have been approved as medical devices through the “Conformité Européenne” (CE) certification. However, due to the unique characteristics of pediatric fractures, it cannot be assumed that an AI model trained on adult fractures will perform equally well in children. Only four programs explicitly include diagnostic capabilities for children: Rayvolve (AZmed), BoneView (Gleamer), SmartUrgences (Milvue), and RBFracture (Radiobotics) [4]. To date, no independent validation for RBFracture regarding fracture detection in children has been presented. Such external validations are highly desirable for AI programs in medical imaging, as manufacturer-associated datasets are often similar to the training data used in software development, thereby limiting generalizability [4, 5]. Moreover, no study with a larger case number has yet demonstrated the added value of AI software for inexperienced physicians in a real-world setting.

Therefore, we aimed to evaluate the AI software RBFracture regarding (1) its stand-alone performance in a pediatric “real-life” cohort and in a set of selected, medicolegally relevant fractures, and (2) the influence of AI assistance on the diagnostic performance of inexperienced residents in the “real-life” cohort.

## Materials and methods

### Cohort

Approval for this retrospective study was obtained from the local ethics committee (357/23-EK). Radiographs of children aged 2 to < 18 years who were referred to the pediatric surgical emergency department of our tertiary center between March 2023 and October 2023 (the “real-life” cohort) were included. Exclusion criteria comprised radiographs of the axial skeleton or skull (both outside the AI software’s intended use), follow-up examinations of known fractures, and non-trauma radiographs (e.g., cases of osteomyelitis).

In addition to the “real-life” cohort, radiographs of children with one of three pediatric-specific fracture types were retrospectively identified from the institutional radiological information system from 2008 to 2023 (the “medicolegal” cohort).

According to van Laer, fractures such as those of the radial condyle, medial malleolus, and proximal tibia are medicolegally relevant if not properly diagnosed and treated. Fractures with dislocation of ≥ 3 mm were excluded from this second group, as such pronounced dislocations are unlikely to be overlooked.

All radiographs were acquired using an Axiom Aristos FX (Siemens) in the Institute for Pediatric Radiology at the study hospital.

### Analysis by the artificial intelligence

**Fig. 1:**
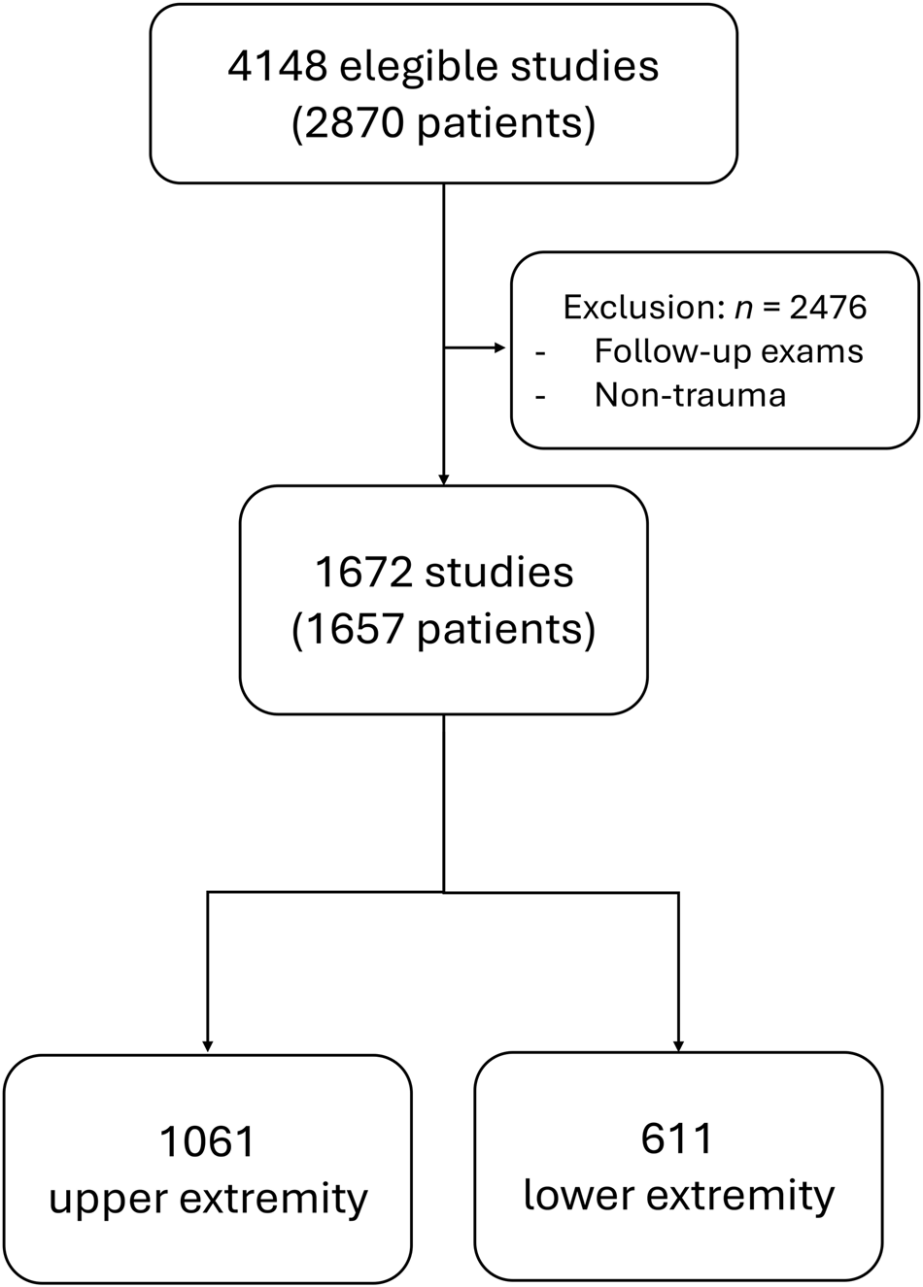
Flowchart of the real-life cohort selection process, showing the inclusion and exclusion criteria applied to identify the final study population of 1673 radiographic studies

All radiographs were transmitted via the Picture Archiving and Communication System (PACS) to a stand-alone server running RBFracture version 1.8.1 (Radiobotics) for offline analysis. RBFracture is based on a deep convolutional neural network using the Detectron2 architecture [6]. The results were returned to the PACS as secondary capture DICOM files (Fig. 1). Potential fractures were highlighted with a rectangular outline in the DICOM images and fractures detected with low confidence were marked with a question mark by the software. Questionable AI findings were conservatively considered as fractures. Each projection was analyzed separately by the AI, independent of other projections. Detection of a fracture in any single projection was considered sufficient for diagnosis. Evaluation was performed primarily study-wise.

### Reference standard for diagnosis

The reference standard for diagnosis was based on (1) the pediatric radiological findings, provided by two board-certified pediatric radiologists (D.G. and F.W.H.) with over 10 years of experience in pediatric traumatology, (2) follow-up radiological images, (3) clinical follow-up, and (4) intraoperative findings when applicable. Inconclusive cases without follow-up radiographs were reviewed by the two additional pediatric radiologists through consensus. Cases where diagnostic uncertainty persisted were excluded from the study (*n* = 14). Radiographs were categorized into the following anatomical regions: shoulder, upper arm, elbow, forearm, wrist, hand, hip, thigh, knee, lower leg, ankle, and foot.

### Performance of human readers

All radiographs of the real-life cohort were reviewed independently and in separate sessions by two pediatric surgical residents working in the pediatric surgery emergency department (A.B. and T.K., with 3 and 6 months of experience in reporting pediatric radiographs, respectively) and a radiology resident working in general radiology on-call (J.P., with 2 years of experience in reporting pediatric radiographs). The readers were blinded to the patient’s identity, any preliminary and follow-up examinations, and the written pediatric radiological findings but not blinded to the patient’s current history and clinical findings to simulate the real-life setting.

All cases were analyzed stepwise. Initially, only the medical history and conventional radiographs were available to the readers. The readers were required to determine the presence and location of a potential fracture. Subsequently, they were presented with the AI’s automated assessment. With this information, the readers were asked to reassess the radiograph. Modifying the initial findings after unblinding to the AI result was not permitted. Additionally, for each case, the readers rated their confidence in the diagnosis both without AI and with AI support on the following scale: 1: uncertain; 2: somewhat unconfident; 3: somewhat confident; 4: certain. Diagnoses were made without a time limit, and to maintain consistent performance, the readers did not receive feedback regarding their performance during the whole study.

### Statistics

A study-wise true-positive was defined as a study in which all actual fractures and possibly more were detected. A study-wise true-negative was defined as a study without fractures in which no fractures were detected. A study-wise false-negative was defined as a study having at least one fracture in which not all actual fractures were detected. A study-wise false-positive (FP) was defined as a study without actual fractures in which fractures were detected. We used these definitions and the usual formulas to define the study-wise sensitivity, specificity, accuracy, positive predictive value (PPV) and negative predictive value (NPV). In addition, we defined the fracture-wise sensitivity as a proportion of fractures correctly identified among all fractures, counting multiple fractures per patient with 95% confidence intervals (CI) (a comprehensive description of the statistics can be found under Supplementary Material 1). Statistical analysis was performed using RStudio (version 2023.06.2, PBC).

## Results

### Cohort

**Fig. 2:**
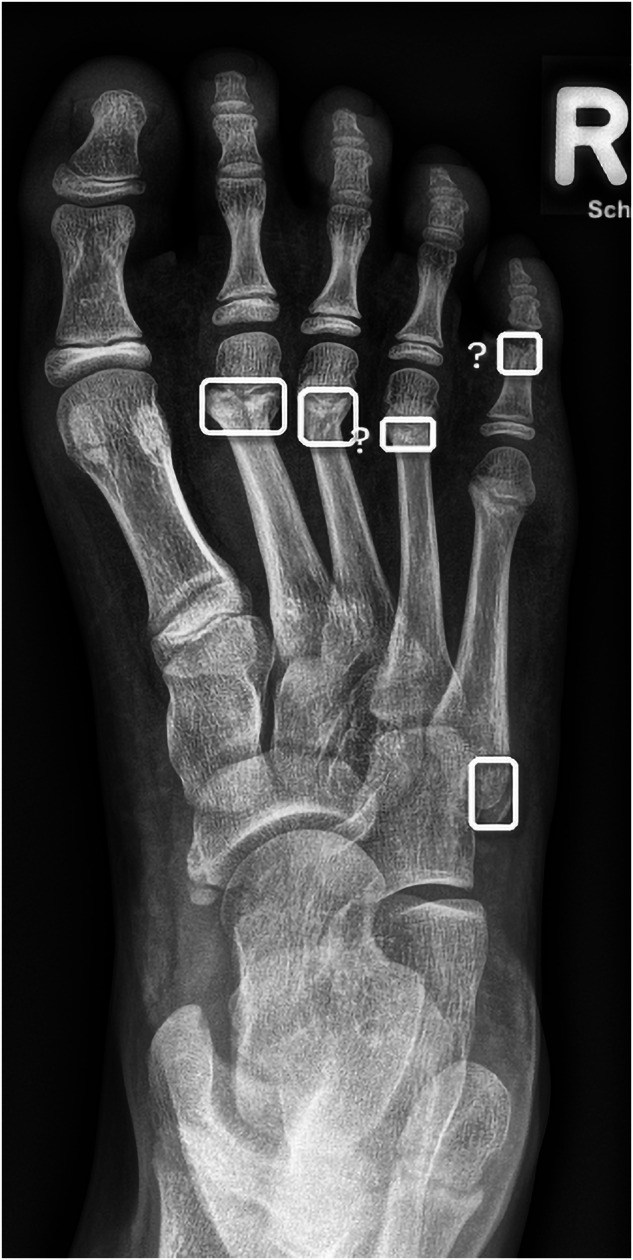
Radiograph of the right foot of an 8-year-old girl showing fractures of the distal second and third metatarsal bones. The AI incorrectly identified a false positive at the apophysis of the fifth metatarsal bone, as well as at the fourth metatarsal and proximal phalanx of the fifth toe. The latter two findings are marked with a question mark due to the AI’s low confidence level

For the real-life cohort, a total of 4148 radiographic studies were identified in the institutional radiological information system, of which 2453 were excluded for follow-up studies of known fractures or non-trauma studies. Thus, 1672 studies were included (Table 1, Fig. 2). At least one fracture was found in 767 of the 1672 studies (46%), resulting in 936 fractures. The median age of the patients was 10.9 years (interquartile range, IQR 7.2–13.7, min.–max. 2.0–18.0). 59% of the patients were male. 63% of the radiographs concerned the upper extremity.

**Table 1:** Demographics of the real-life cohort

Age (years)	10.9 (7.2–13.7)
Gender	59% male, 41% female
Number of patients	1657
Number of studies	1672
Upper extremity	1061
Lower extremity	611
Number of fractures	936

### Stand-alone performance

**Fig. 3:**
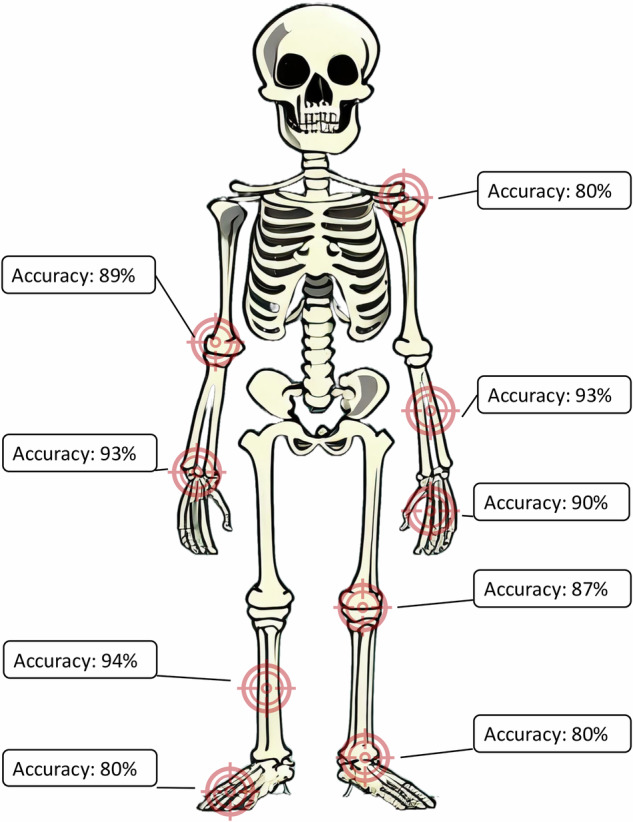
Accuracy of the AI across different body regions based on 1673 radiographic studies. Due to small sample sizes, the upper arm, hip, and thigh were not analyzed separately

Depending on the body region, AI achieved a patient-wise sensitivity ranging from 74 to 98% and specificity ranging from 61 to 100% (Table 2). Accuracy varied from 80 to 94% (Fig. 3). In the “real-life” cohort, the NPV of 93% was significantly higher than the PPV of 82% (*p* < 0.05). 60 of 936 fractures (6.4%) were missed by the AI (Supplementary Table 1). More than half of those missed fractures consisted of small ligamentous avulsions (*n* = 21) or torus fractures (*n* = 10). The performance of AI in small children below 5 years of age was not significantly lower than above 5 years (Supplementary Table 2).

**Table 2:** Performance parameters for detecting pediatric fractures by body region (95% confidence interval in parentheses)

	*n*	Sensitivity (%)	fw-Sensitivity (%)	Specificity (%)	Accuracy (%)	PPV (%)	NPV (%)
Shoulder	94/55	93 (85–98)	93 (85–98)	61 (45–76)	80 (71–87)	78 (66–87)	85 (70–97)
Elbow	180/95	89 (82–95)	90 (83–95)	90 (83–96)	89 (84–94)	90 (83–96)	89 (82–95)
Forearm	85/104	98 (94–100)	99 (96–100)	78 (59–95)	93 (87–98)	92 (85–99)	95 (80–100)
Wrist	350/324	97 (94–99)	98 (96–99)	85 (78–91)	93 (90–95)	92 (88–95)	94 (89–98)
Hand	346/177	89 (84–94)	90 (86–94)	91 (86–95)	90 (87–93)	90 (85–94)	90 (86–95)
Knee	85/12	75 (44–100)	75 (44–100)	89 (81–96)	87 (80–94)	53 (25–78)	96 (90–100)
Lower leg	18/13	92 (73–100)	92 (75–100)	100 (54–100)^a^	94 (83–100)	100 (72–100)^a^	86 (50–100)
Ankle	248/52	74 (61–86)	77 (64–88)	81 (76–87)	80 (75–85)	47 (36–59)	93 (89–97)
Foot	254/101	86 (80–93)	87 (81–94)	77 (70–83)	80 (76–85)	69 (60–76)	90 (85–95)
Overall	1672/933	92 (90–94)	94 (92–95)	83 (81–86)	87 (86–89)	82 (79–84)	93 (91–94)

The upper arm, hip, and thigh were not analyzed separately due to the small number of cases (*n* = 12)

*PPV* positive predictive value, *NPV* negative predictive value, *fw* fracture-wise, *n* number of patients; number of cases/number of fractures

^a^ The Clopper-Pearson exact method was used to calculate the 95% confidence intervals

The most frequent body region with FP findings by the AI included the apophysis of the 5th metatarsal bone (16%) and normal variants of the toes (13%) (Table 3). The most common cause of FP findings involved regions around the growth plates, such as epiphyses, apophyses, and other bone nuclei (Supplementary Data 2 and Supplementary Table 3). 90% of FP findings were labeled as “low-confidence” by the AI.

**Table 3:** Common locations for false-positive findings by the AI (*n* = 200)

	Frequency (%)
Metatarsal	16% (*n* = 32)
Toe	13% (*n* = 25)
Ankle (medial)	10% (*n* = 20)
Finger	10% (*n* = 19)
Ankle (lateral)	6% (*n* = 12)
Ulna (distal)	6% (*n* = 12)
Other	40% (*n* = 79)

*n* number of cases

**Fig. 4:**
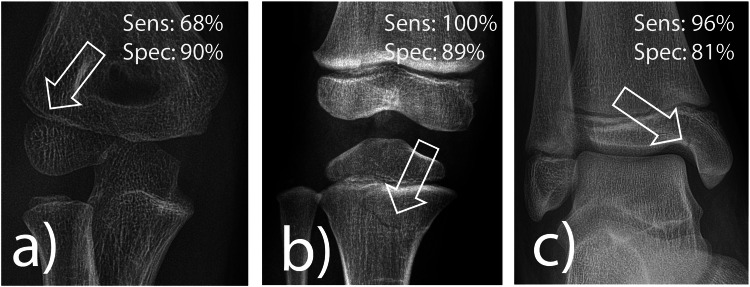
Frequently missed fracture entities with medicolegal significance according to van Laer. **a** Radial condyle fracture, (**b**) fracture of the proximal tibia, and (**c**) fracture of the medial malleolus. The lucency marked by an arrow indicates the fracture. Sens, sensitivity; Spec, specificity

In the cohort of medicolegal relevant fractures, AI demonstrated the best combination of sensitivity (100%) and specificity (86%) for proximal metaphyseal tibial fractures, followed by medial malleolus fractures (sensitivity 96%, specificity 95%) (Table 4 and Fig. 4). In contrast, sensitivity for radial condyle fractures tended to be lower at 68% (not significant).

**Table 4:** Performance of the artificial intelligence software in detecting selected medicolegally relevant fracture types

	*n*	Sensitivity (%)	Specificity (%)
Radial condyle	25	68 (46–85)	90 (83–96)
Medial malleolar	23	96 (78–00)	81 (67–87)
Proximal tibia	25	100 (86–100)	89 (81–96)

Sensitivity was determined in a selected cohort containing only fractures, while specificity was estimated using pooled normal findings from the corresponding regions of the real-life cohort

*n* number of cases; 95% confidence interval in parentheses

### Impact on first-line physicians

**Fig. 5:**
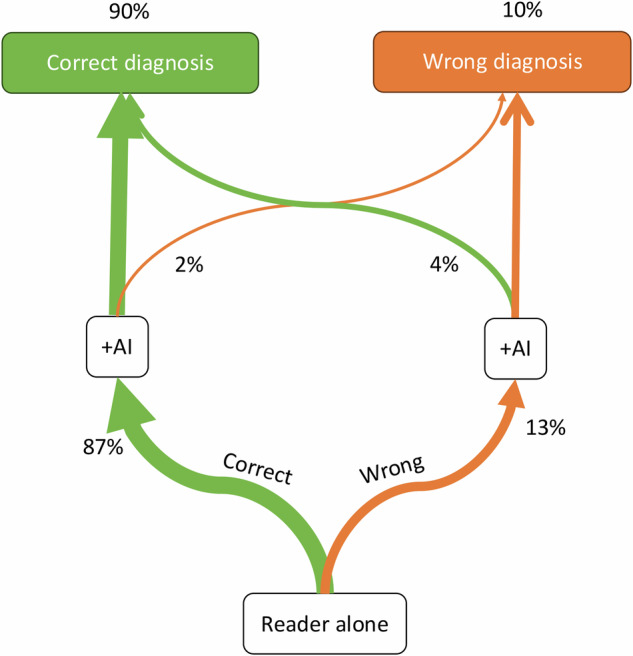
Impact of AI on the initial diagnoses made by inexperienced residents. The accuracy improved from 87.3 to 89.9% with the addition of AI. However, in 1.5% of cases, the AI incorrectly led to a change from a previously correct diagnosis

Non-expert readers correctly diagnosed 87.5% of patients without AI assistance (Fig. 5). There was no significant difference between first-year pediatric surgical and radiological residents (*p* < 0.05). The mean number of missed fractures of the readers was reduced by AI from 129 (13.8% of all fractures) to 100 (10.7% of all fractures), resulting in a decrease of missed fractures of 22%.

The addition of AI corrected the reader’s initial wrong patient-wise diagnosis in 4.1%, whereas in 1.5%, the reader’s initial right diagnosis was wrongly rejected. This resulted in a net gain of 2.6% in correct diagnoses (90.1%) (*p* < 0.05). With AI assistance, patient-wise sensitivity increased from 83.7 to 87.3% and specificity from 90.7 to 92.4% (both *p* < 0.05) (Table 5).

**Table 5:** Specificity and sensitivity (with absolute difference) for fractures in the real-life cohort as assessed by human readers, with and without the assistance of AI

	Sensitivity (%)			Specificity (%)		
	−AI	+AI	Delta	−AI	+AI	Delta
Reader 1	83.1	84.9	1.9	92.9	96.3	3.3
Reader 2	89.9	93.4	3.5	84.7	86.4	1.8
Reader 3	78.1	83.8	5.7	94.5	94.6	0.0
Overall	83.7 (82–86)	87.3 (86–89)	3.7 (3–5)	90.7 (90–92)	92.4 (91–94)	1.7 (1–3)

“Overall” represents the average performance of the readers

Values may not add up due to rounding. Numbers in parentheses are 95% confidence intervals

*+AI* with AI assistance, −*AI* without AI assistance, *Delta* absolute difference

Diagnostic confidence increased unblinding to the AI results (mean change across the readers 0.18, *p* < 0.001). Diagnostic confidence was high when readers selected the correct diagnosis, both with and without AI assistance (means between 3.24 and 3.84), and lower when choosing the wrong diagnosis (means between 2.52 and 3.04). In cases where AI led to a change in diagnosis, initial confidence was low (means between 1.43 and 2.06) but improved in 2 out of 3 readers when AI led to the correct decision (mean changes: 0.40, *p* = 0.02; 0.30, *p* = 0.03; −0.07, *p* = 1 for the 3 readers, respectively). Conversely, the confidence decreased significantly in 2 readers when AI led to a change from a correct to an incorrect diagnosis (mean changes: −0.11, *p* = 0.29; −0.81, *p* = 0.02; −0.55, *p* = 0.04 for the 3 readers, respectively). All readers showed increased diagnostic confidence in cases where both the unaided and aided diagnoses were correct (mean changes: 0.23; 0.08; 0.36, all *p* < 0.001).

## Discussion

This study evaluated the performance of a commercially available AI-supported software for fracture detection in children and adolescents, as well as its impact on the diagnostic accuracy and confidence of inexperienced residents in a real-life clinical setting. To date, this is the largest study of its kind. The AI demonstrated a good stand-alone performance; however, the improvement in the diagnostic accuracy of inexperienced physicians when using this AI assistance was limited.

### Stand-alone performance

In our cohort, AI achieved a sensitivity of 92%, specificity of 83%, and diagnostic accuracy of 87%. In the real-life cohort, a particularly high number of FP findings was identified at the apophysis of the 5th metatarsal bone. Empirically, this region often presents challenges in differentiating between a fracture and an apophysis in children, particularly for inexperienced physicians. Additionally, the often-irregular shape of toes was frequently misinterpreted as a fracture—a distinction that, in our experience, is particularly difficult for beginners. As anticipated, the physis gap, resembling a fracture line or avulsion, accounted for more than half of all FP findings, significantly contributing to misdiagnoses.

The stand-alone performance of the AI in our study is comparable to that reported in the few peer-reviewed studies of (predominantly other) AI software solutions in children [7–12]. However, the validity of such comparisons across different software tools and cohorts is limited. For example, it is important to consider whether the cohort includes polytrauma patients, as their absence could result in an underrepresentation of more severe (and typically easier to detect) fractures. Additionally, the age distribution within the cohort is crucial, as certain fracture types are age-specific (e.g., toddler fractures in infants or triplane fractures in adolescents).

For RBFracture, the AI software used in this study, only one peer-reviewed publication to date including a small set of children. In this manufacturer-associated study by Bachmann et al a stratified cohort of 98 children (generously defined as individuals up to 21 years of age) was employed [9]. They reported an exceptionally high sensitivity of 100% (95% CI: 100–100%) and a moderate specificity of 79%. The higher sensitivity compared to our study and other AI-based programs could be attributed to their small sample size or potentially higher median patient age, which may have resulted in a greater prevalence of closed growth plates—the most common source of error in pediatric fracture detection.

### Fractures of particular medicolegal relevance

Trauma-related complications have been described as the leading cause of malpractice lawsuits against pediatric surgeons in some countries, accounting for 27% of all cases [13]. A study by Vinz et al retrospectively analyzed 189 cases investigated before a court of arbitration for potential treatment errors in pediatric fractures [14]. They found that 67% involved medical malpractice, often due to misinterpretation of radiographs, with 44% of these patients suffering permanent damage due to incorrect treatment.

According to van Laer, five trauma sequelae have been identified that are often overlooked in children and, if not treated appropriately, can potentially lead to permanent damage, such as valgus deformity of the leg or growth disturbances at the elbow [15]. These trauma sequelae include three types of fractures—non-displaced radial condyle fractures, proximal tibia fractures, and medial malleolus fractures—and two malalignments, which are not part of this fracture study.

For these three fracture types, characterized by subtle fracture morphology but high clinical impact, achieving the highest possible AI sensitivity is crucial. In our study, the AI demonstrated a sensitivity of 100% for proximal tibia fractures, 96% for medial ankle fractures, but only 68% for radial condyle fractures. This latter finding is concerning, as the elbow is a frequent site of medical errors in pediatric fractures, accounting for 77% of all cases, according to Vinz et al [14]. Consequently, more intensive AI training focused on pediatric elbow fractures, particularly this type of subtle fracture, would be highly beneficial.

### Impact on inexperienced physicians

According to the hierarchical efficiency model for AI software in imaging proposed by Leeuwen et al, pure stand-alone performance represents only the second-lowest level [16]. The next level involves the impact of the software on the physician’s performance, which we also evaluated. To the best of the authors’ knowledge, this is the first study to provide such evidence in a real-life pediatric cohort. In our study, AI improved initially incorrect diagnoses in 4.1% of cases. However, it is noteworthy that in 1.5% of cases, AI led to the incorrect alteration of an initially correct diagnosis, resulting in a net improvement of 2.6% in reader accuracy due to AI.

Apart from our study, only two other studies have investigated the added value of AI on human readers. Both were not independent of the manufacturer and involved stratified cohorts based on fracture location, rather than a real-life cohort. In the aforementioned study by Bachmann et al [9], using the AI software RBFracture, the proportion of missed fractures was assessed instead of accuracy. With AI assistance, the rate of missed fractures decreased by 29%, more than our study’s rate (21%). This difference may primarily be due to the fact that the readers in that study were mostly paramedical staff, who likely have a lower performance level in initial fracture detection compared to physicians. For example, the rate of missed fractures among advanced trauma nurses was 79.0% (compared to 14.3% in our study), which is an unacceptably high figure in practice. Additional factors that may contribute to the higher reduction in missed fractures in that study include cohort stratification, the lack of clinical history available to the readers, the potentially higher sensitivity of the software version used, and the very small proportion of children in the analyzed cohort.

In the equally mentioned study by Nguyen et al, incorporating the AI software BoneView improved the sensitivity of junior radiologists by 10.3% and their specificity by 0.7% (3.6% and 1.7%, respectively, in our study) [17]. Whether this difference is due to the varying training levels of the readers, the stratified patient dataset, or the differing performance of the software used remains speculative.

While no further studies have specifically examined AI’s impact on human reader performance in children and adolescents, similar research exists for stratified adult cohorts. Guermazi et al reported that the addition of the BoneView software improved the sensitivity of emergency physicians by 9.9% and the specificity by 3.4% [18]. In Duron et al, the same AI software increased sensitivity by 7.5% and specificity by 4.1% [19].

Whether purchasing fracture detection software is worthwhile depends not only on its stand-alone performance and its impact on human readers but also on a complex question that cannot be answered unequivocally: What level of economic investment is justified for a defined increase in performance and a potential improvement in patient safety? In clinical practice, AI-based software should aim to (1) improve patient safety by acting as a “second pair of eyes” (known also as augmented AI [5]), helping to prevent harm caused by undiagnosed fractures or unnecessary immobilization, (2) reduce the number of return visits due to initial misdiagnoses, (3) assist in training less experienced junior doctors, and (4) enable the automatic triage of patients to ensure timely and appropriate care. Despite the limited overall increase in accuracy, based on our experience with fracture detection AI software, even experienced pediatric radiologists benefit from this “second pair of eyes,” leading to a reduction in satisfaction-of-search errors. This is especially true when fractures are located in unexpected areas, such as a partially imaged bony ligament tear in the upper ankle joint, even though the forefoot was the primary focus.

Future studies, preferably prospective and multicenter, should aim to achieve the next highest level of evidence: the effect on treatment outcomes or follow-up examinations, impact on quality of life, morbidity, survival, and the cost-effectiveness in terms of quality-adjusted life years and incremental cost per quality-adjusted life years [16].

### Limitations

In addition to its retrospective, monocentric nature, this study has some other limitations. For example, updates to the AI software used may have been published between the start of the study and its publication, which could yield different results. Due to the large cohort, a certain learning effect among readers cannot be ruled out despite blinding to the gold standard. Our real-life design, in which the diagnosis was established first without AI and immediately followed by the same case with AI, could have resulted in readers being less willing to change their initial diagnosis. However, this approach most accurately reflects the decision-making process in real-life scenarios. Additionally, the performance of initially inexperienced doctors would likely show a steep increase during a potential wash-out phase of several months, an effect that was largely circumvented by our study design. Finally, an expert consensus as the ground truth is not perfect, and this applies not only to our study but also to all comparable studies (and to the AI training data as well). Furthermore, pediatric radiologists have demonstrated high diagnostic accuracy in evaluating pediatric trauma cases [10].

## Conclusion

In summary, the AI software showed good stand-alone performance in a pediatric real-life cohort. In our university hospital setting of a tertiary center, inexperienced residents benefit in a limited proportion of cases. This benefit must be weighed against the economic cost.

## Supplementary information

ELECTRONIC SUPPLEMENTARY MATERIAL

## Abbreviations

AI: Artificial intelligence
CI: Confidence interval
FP: False-positive
NPV: Negative predictive value
PACS: Picture Archiving and Communication System
PPV: Positive predictive value
