# Supplementary figures and images for: Real-life benefit of artificial intelligence-based fracture detection in a pediatric emergency department
Source: Eur Radiol. 2025 Apr 7;35(10):5881–90. doi: 10.1007/s00330-025-11554-9 (PMC12417293; doi:10.1007/s00330-025-11554-9)

False positives

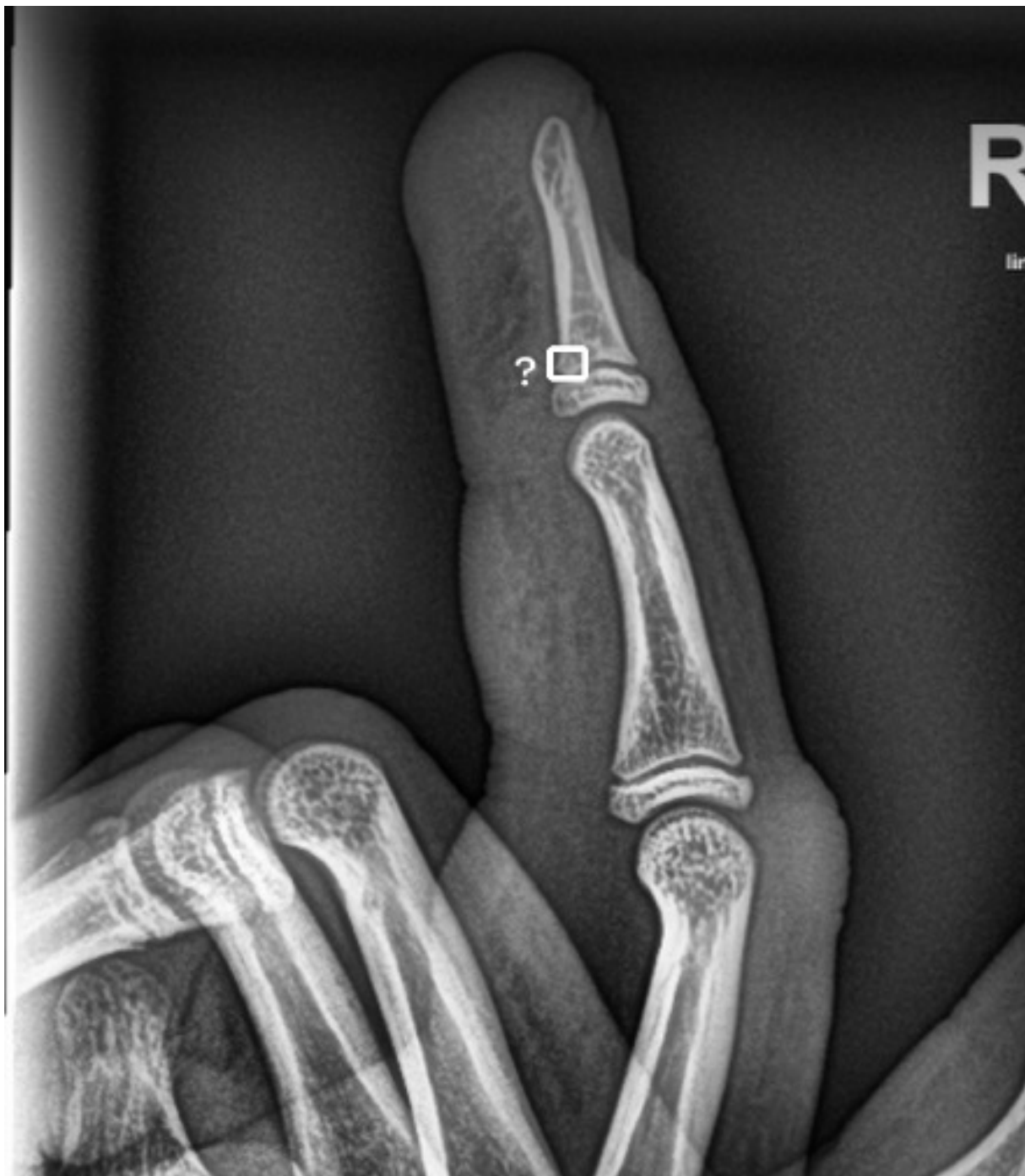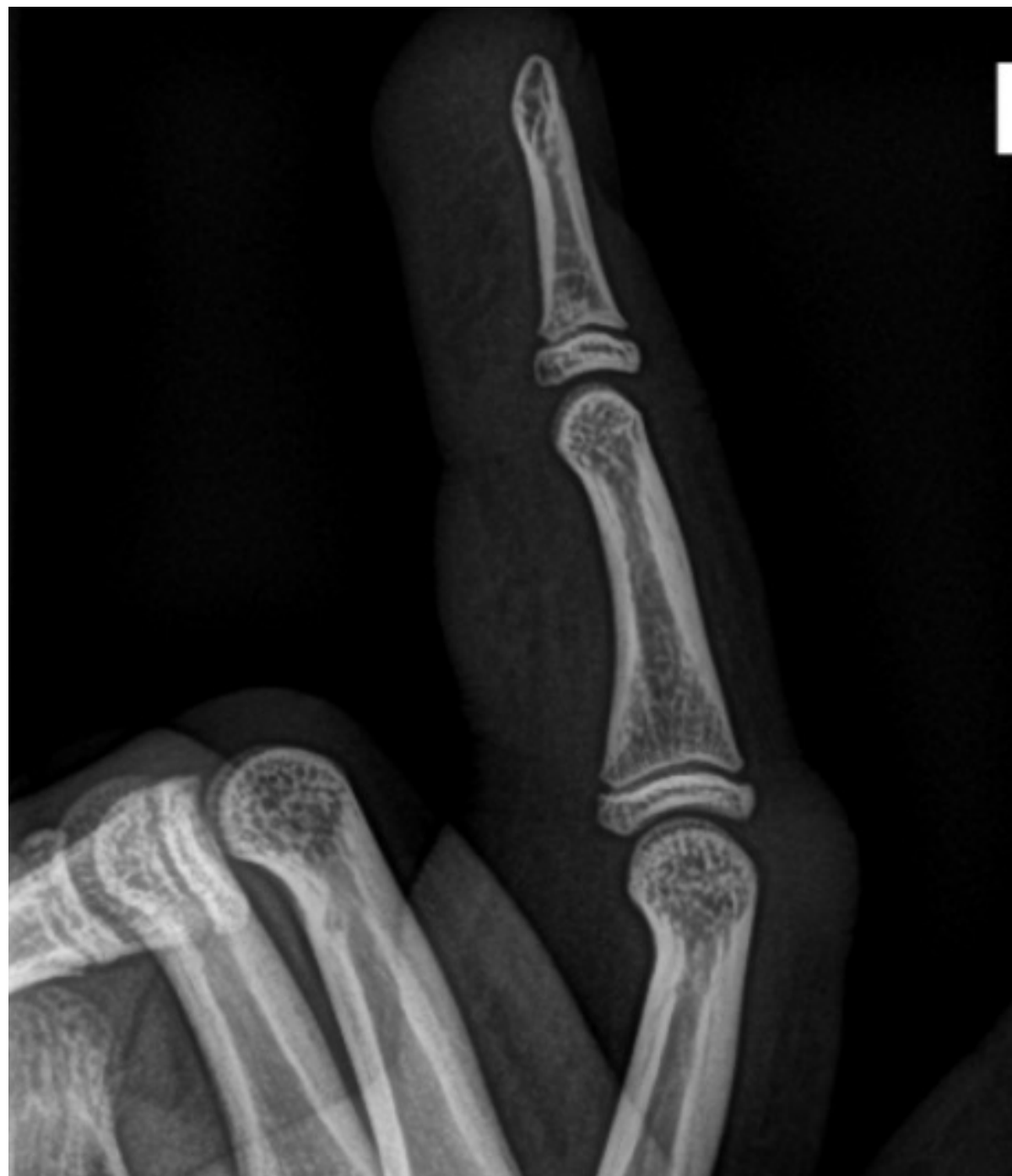

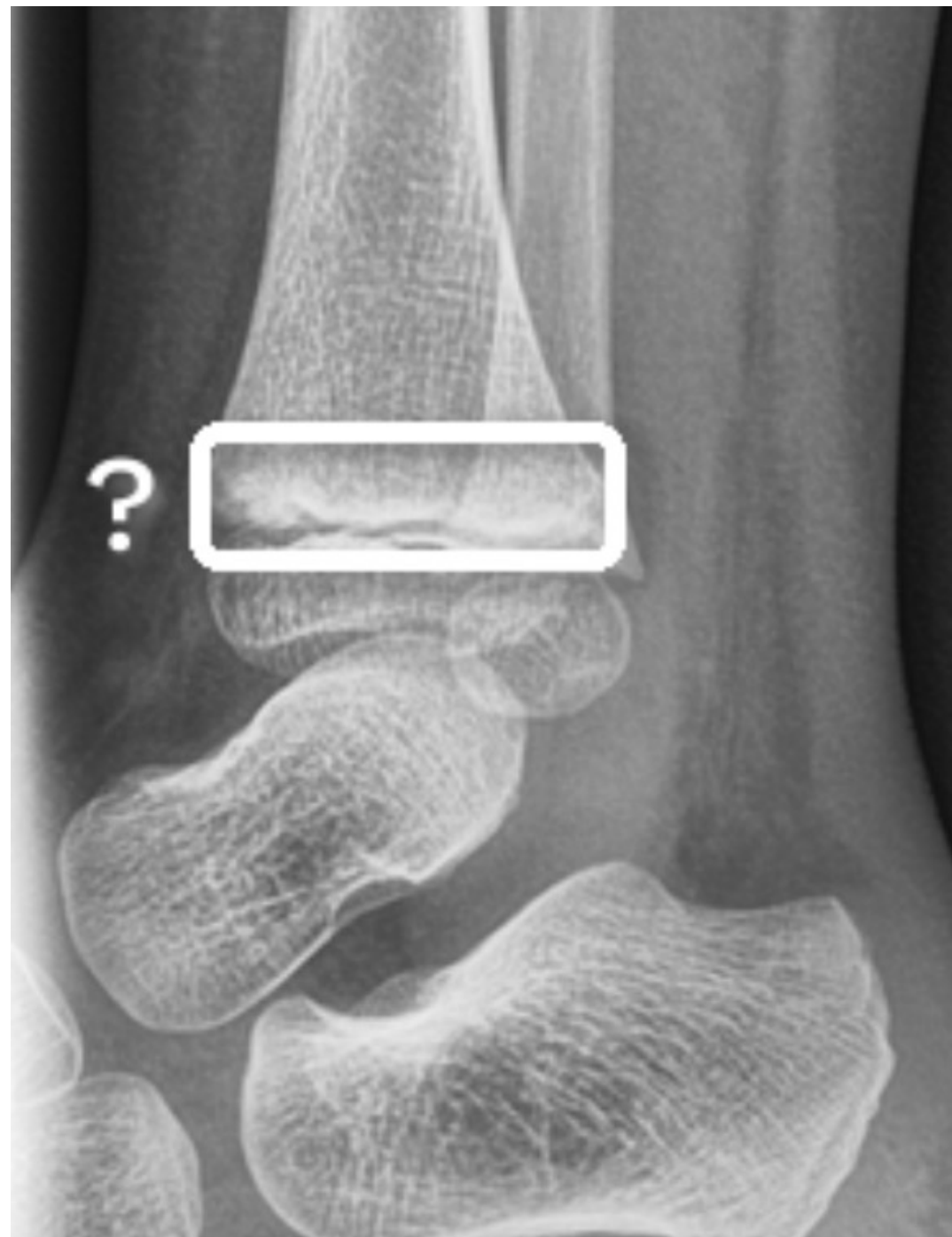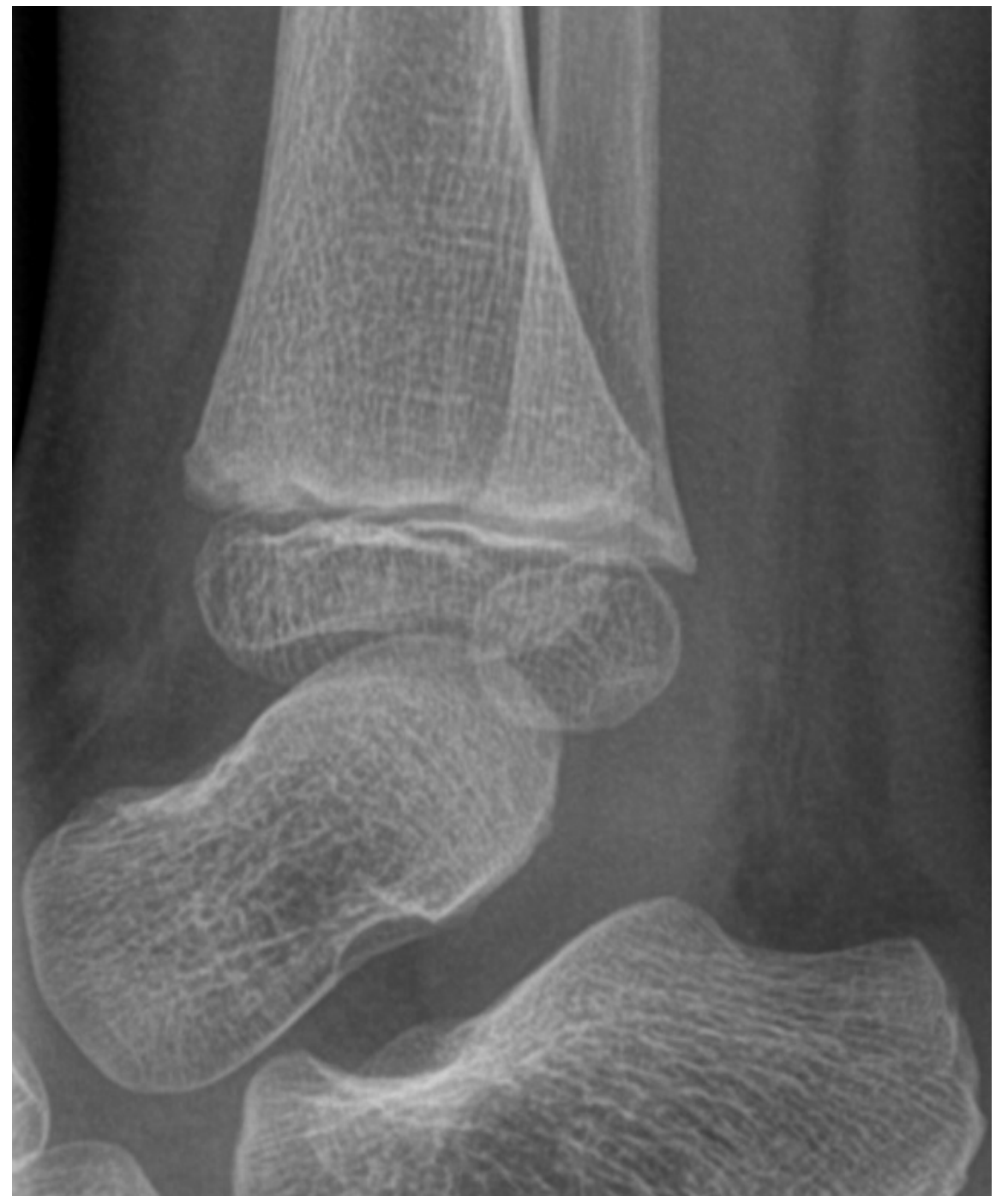

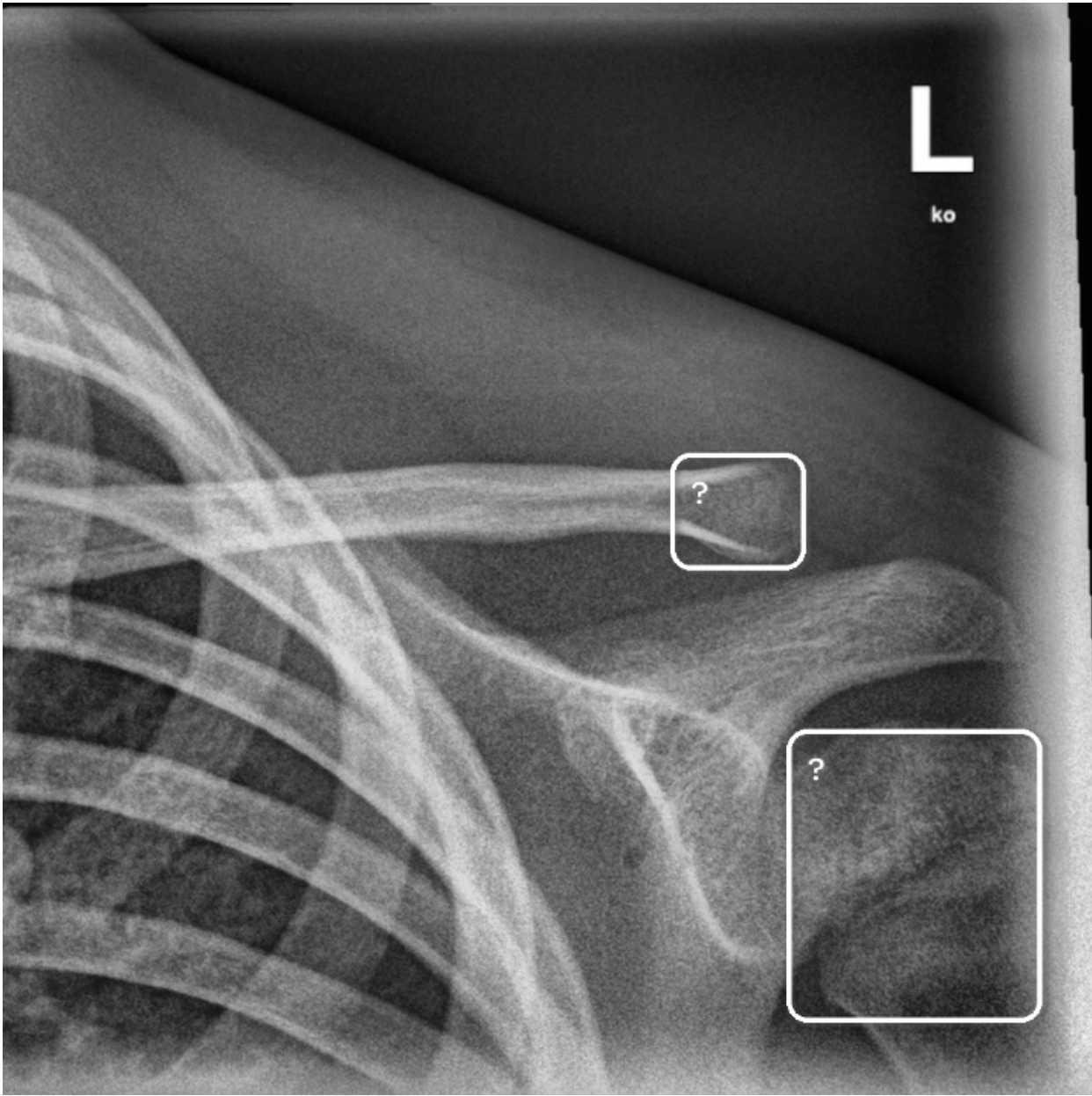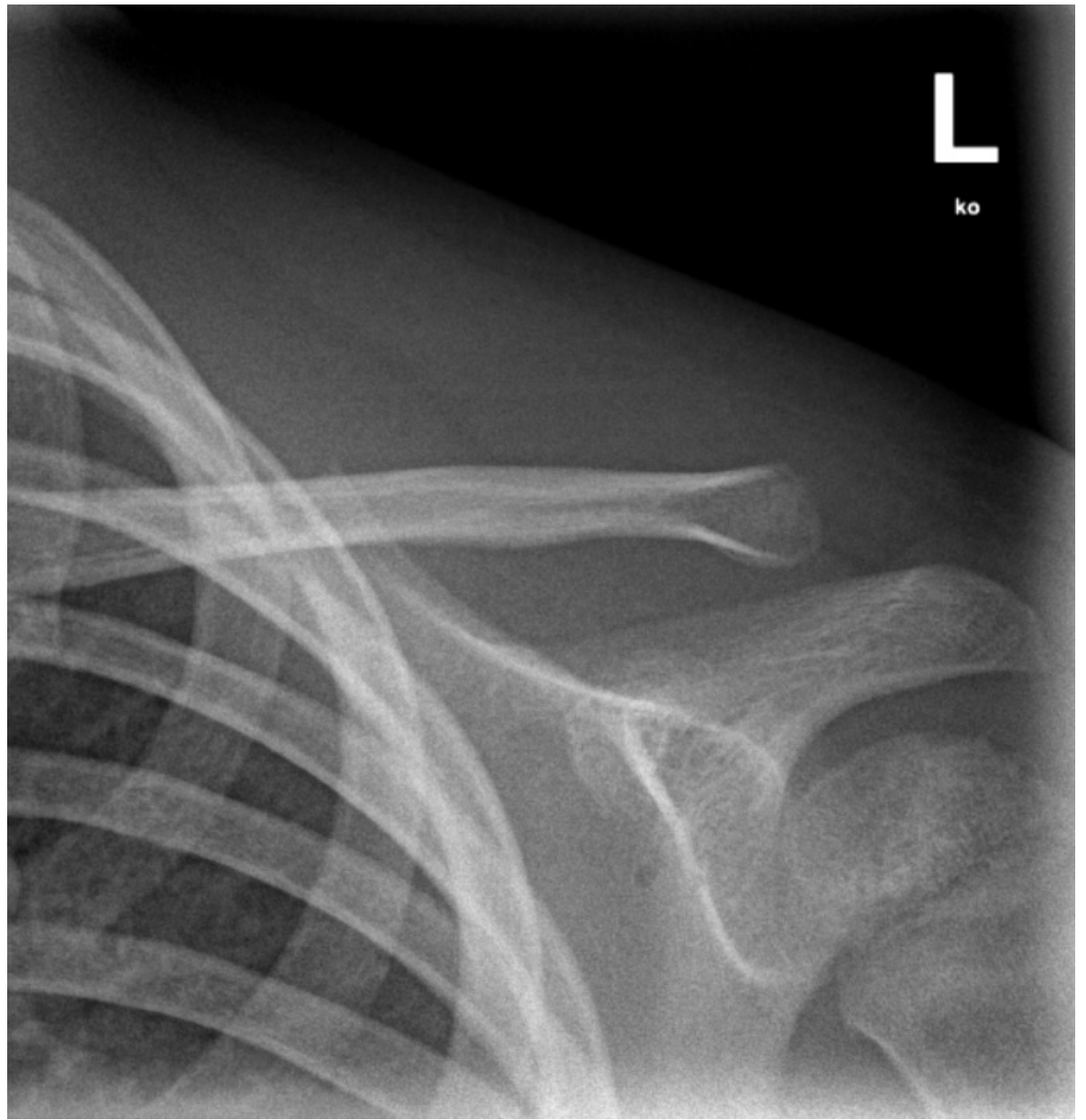

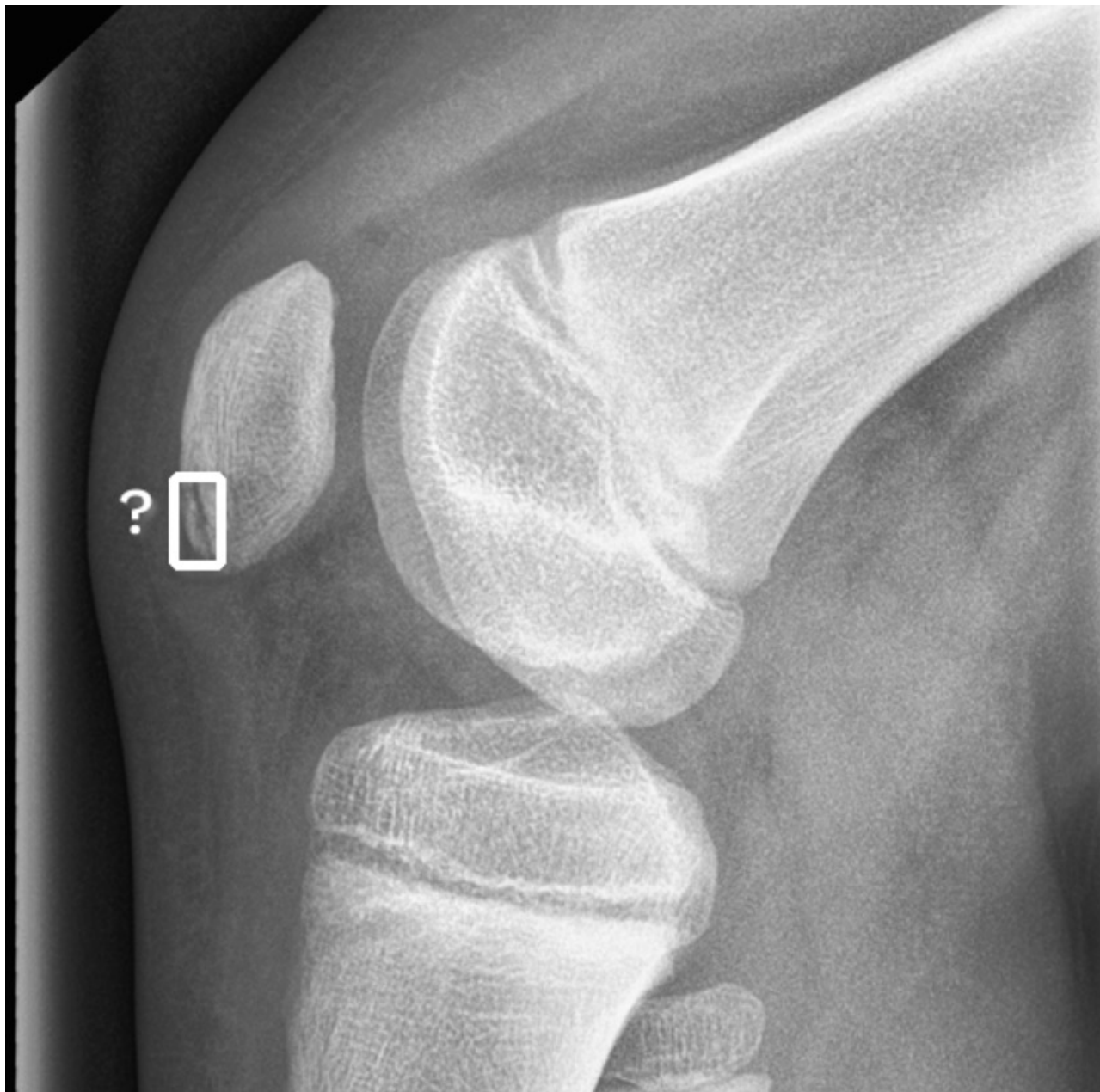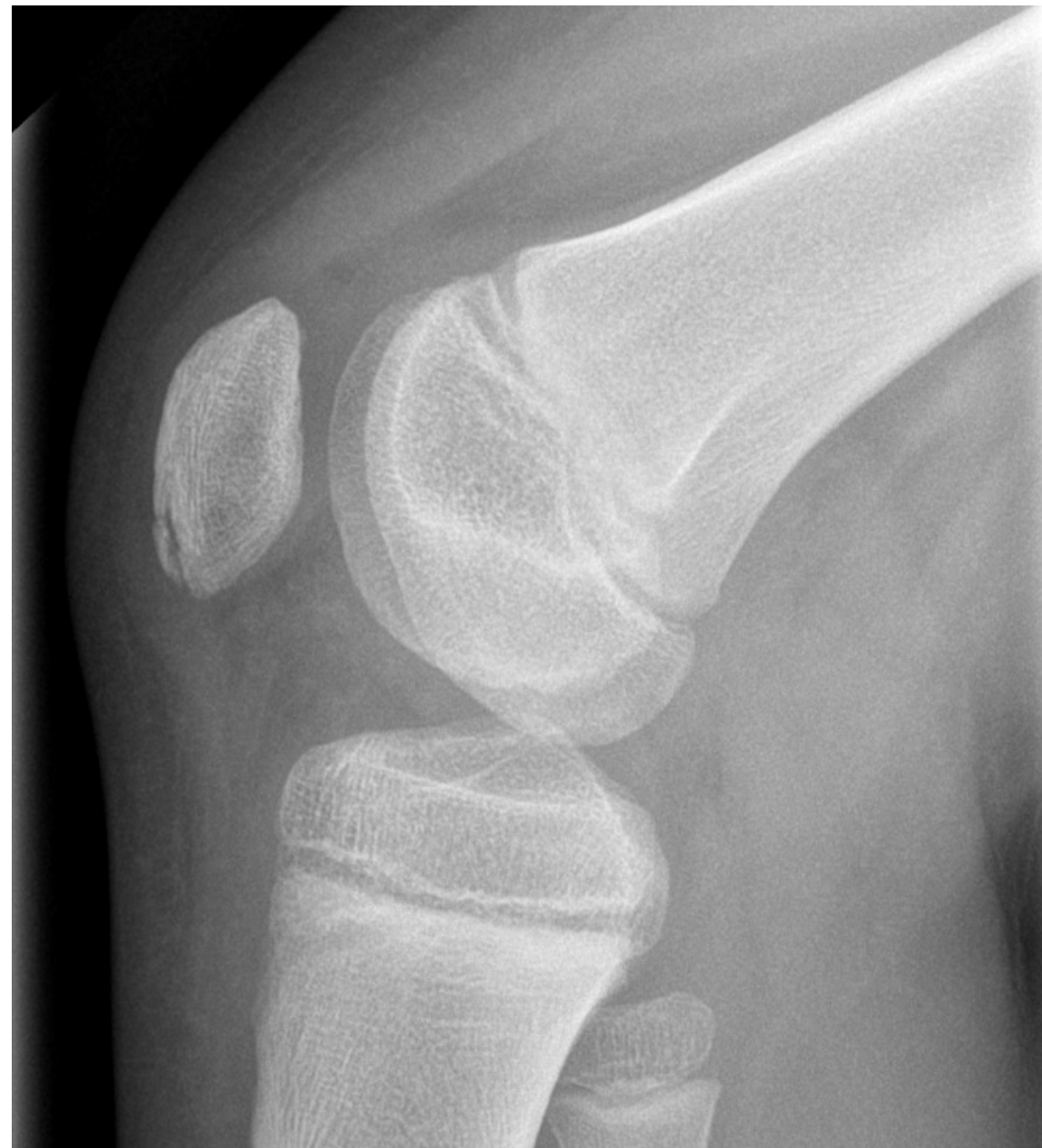

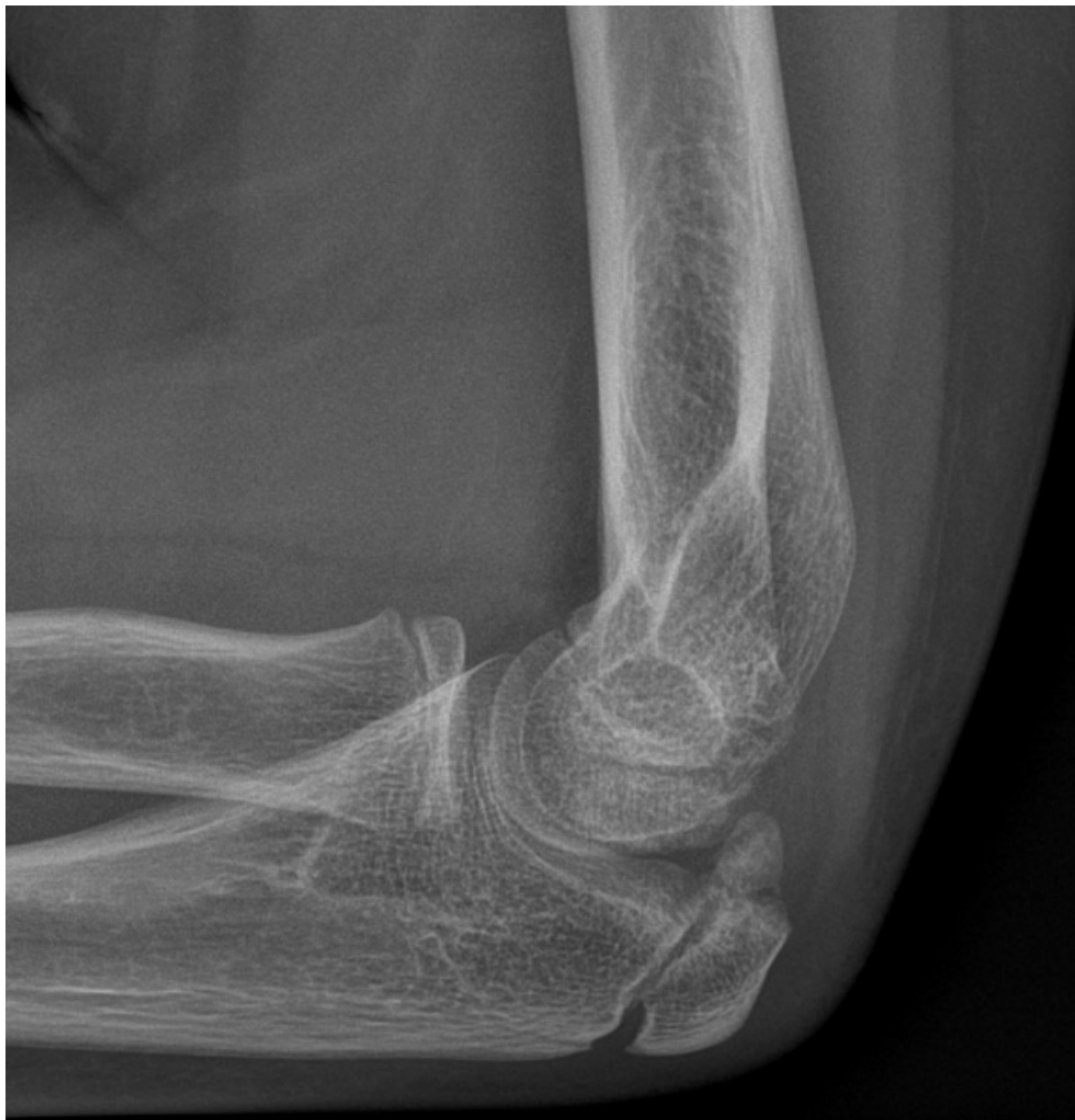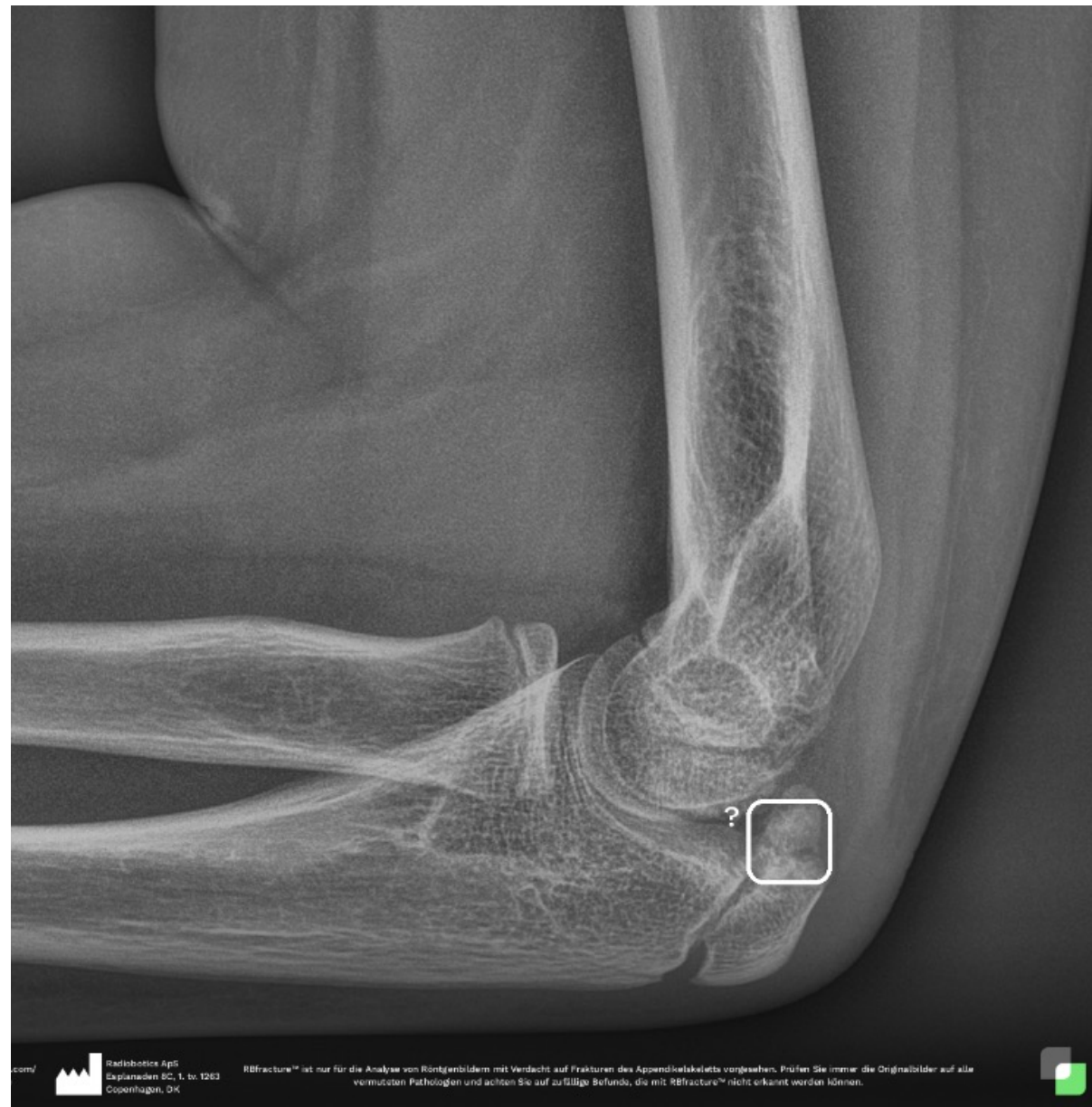

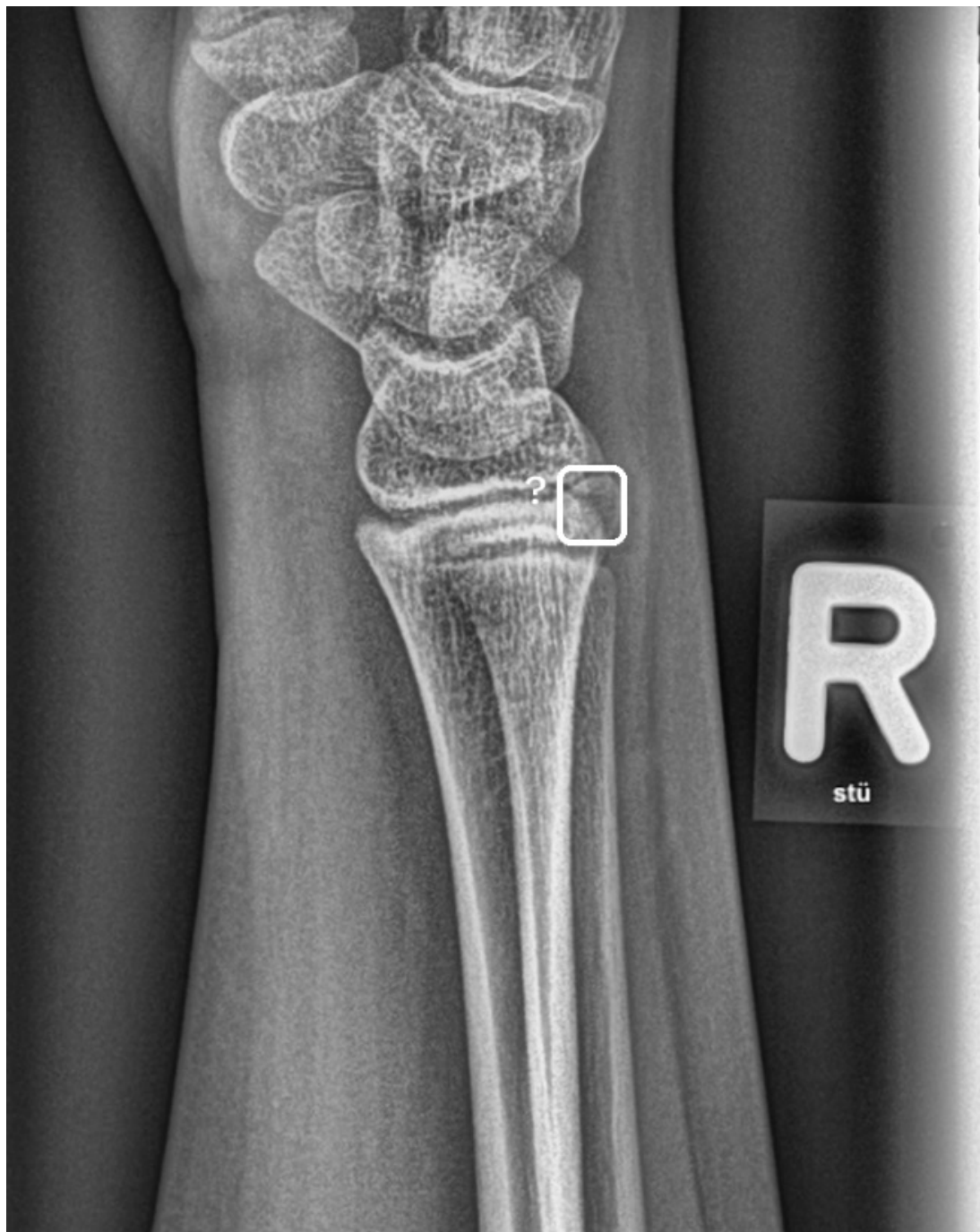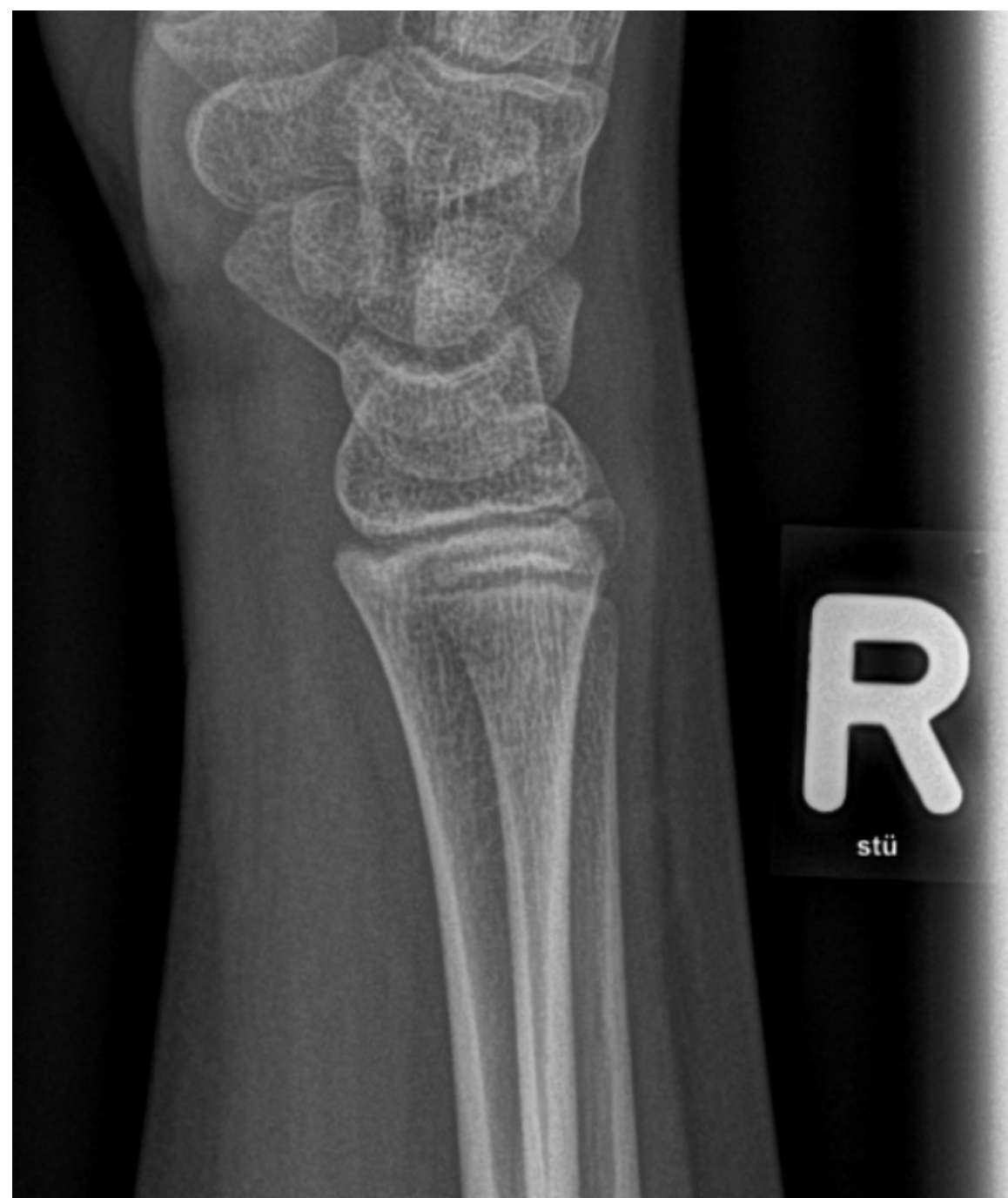

False negatives

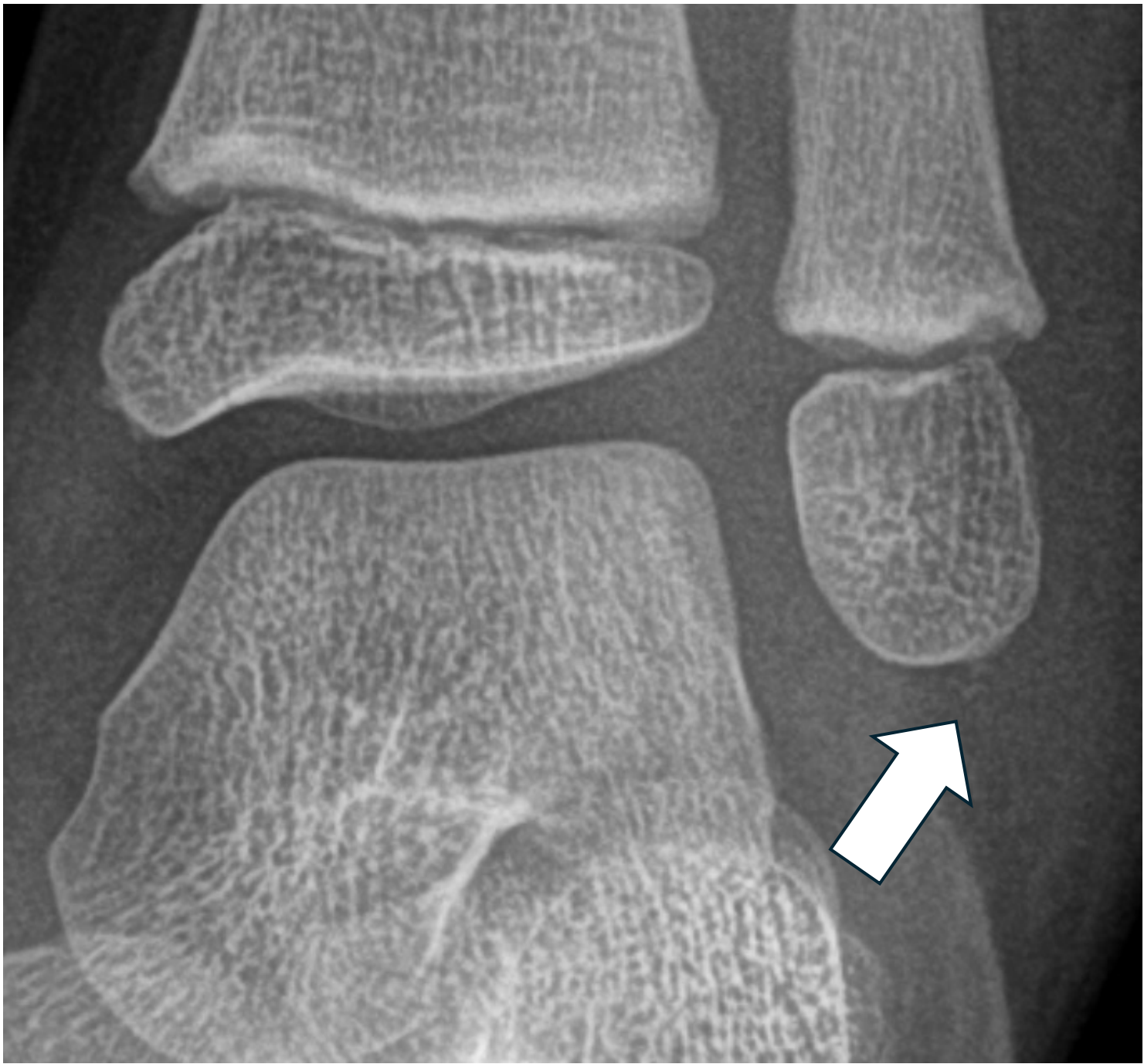

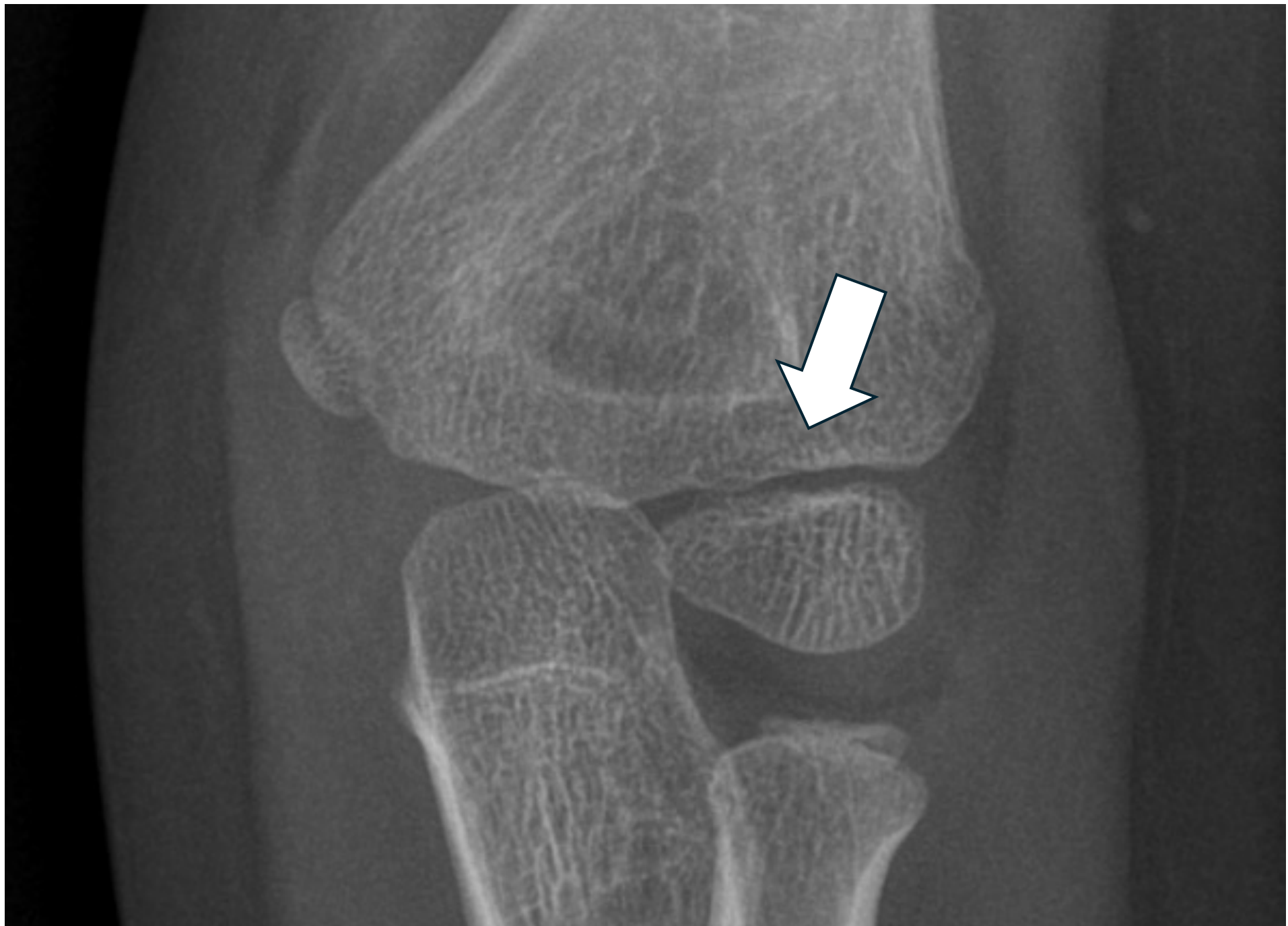

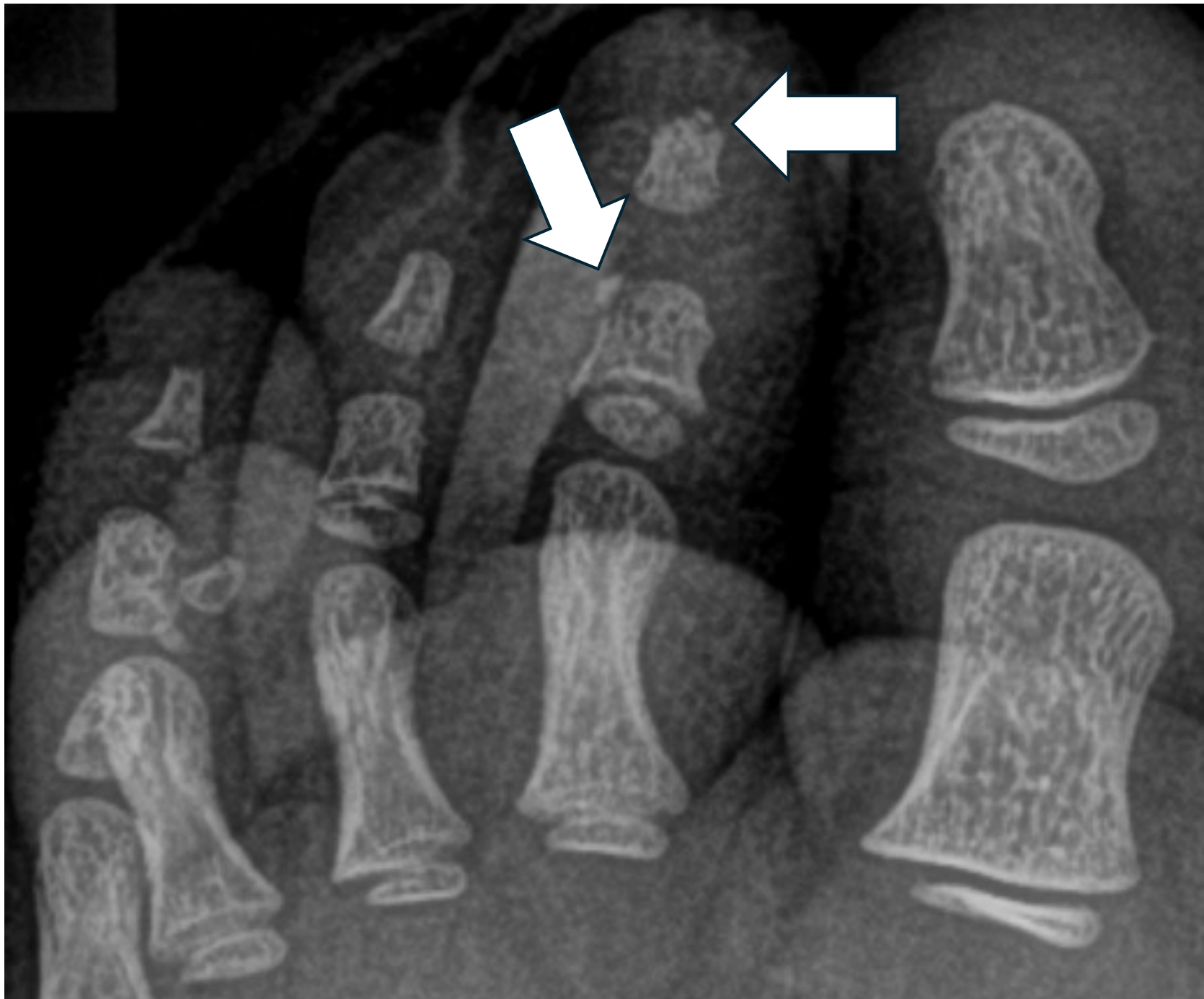

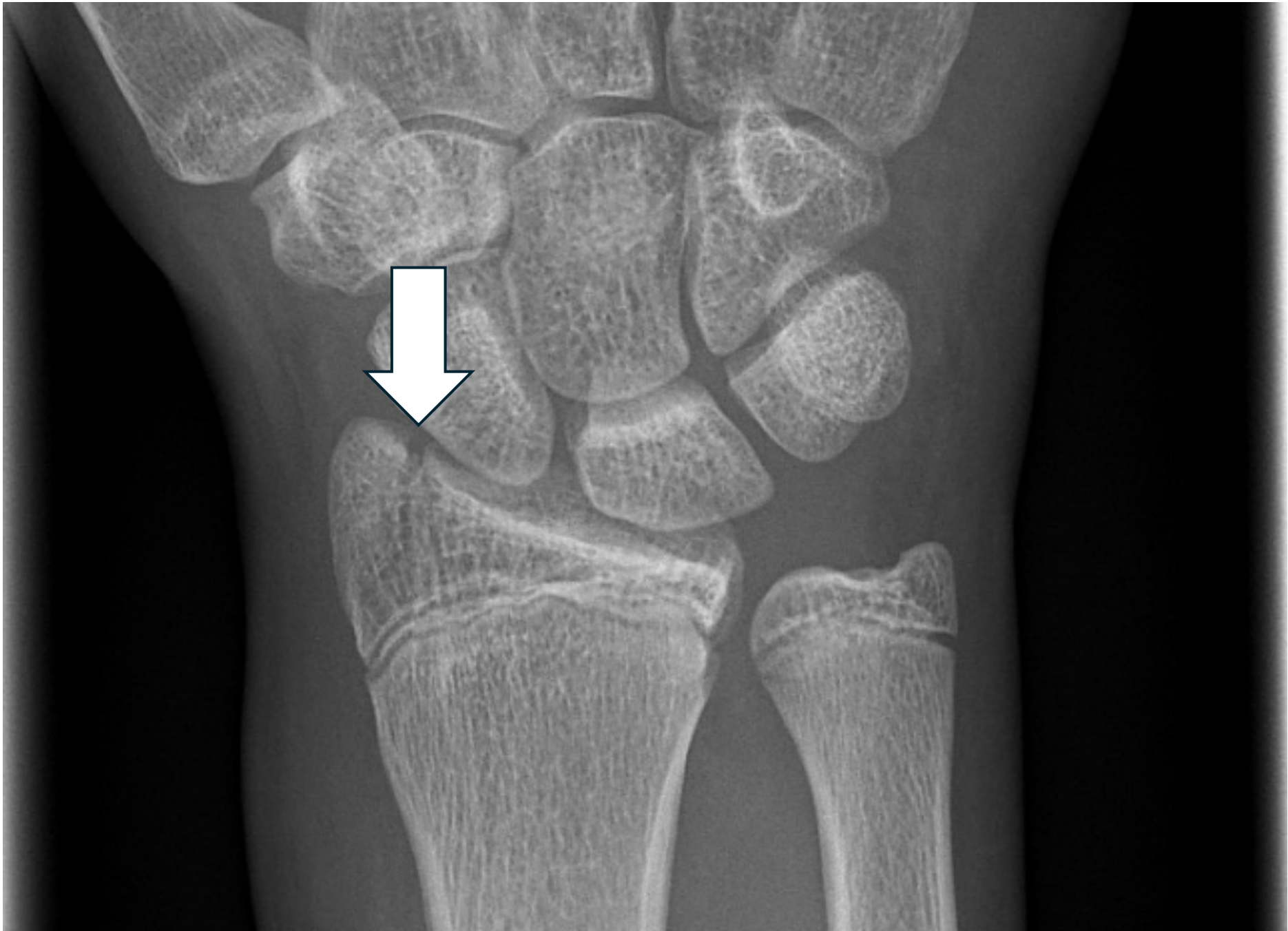

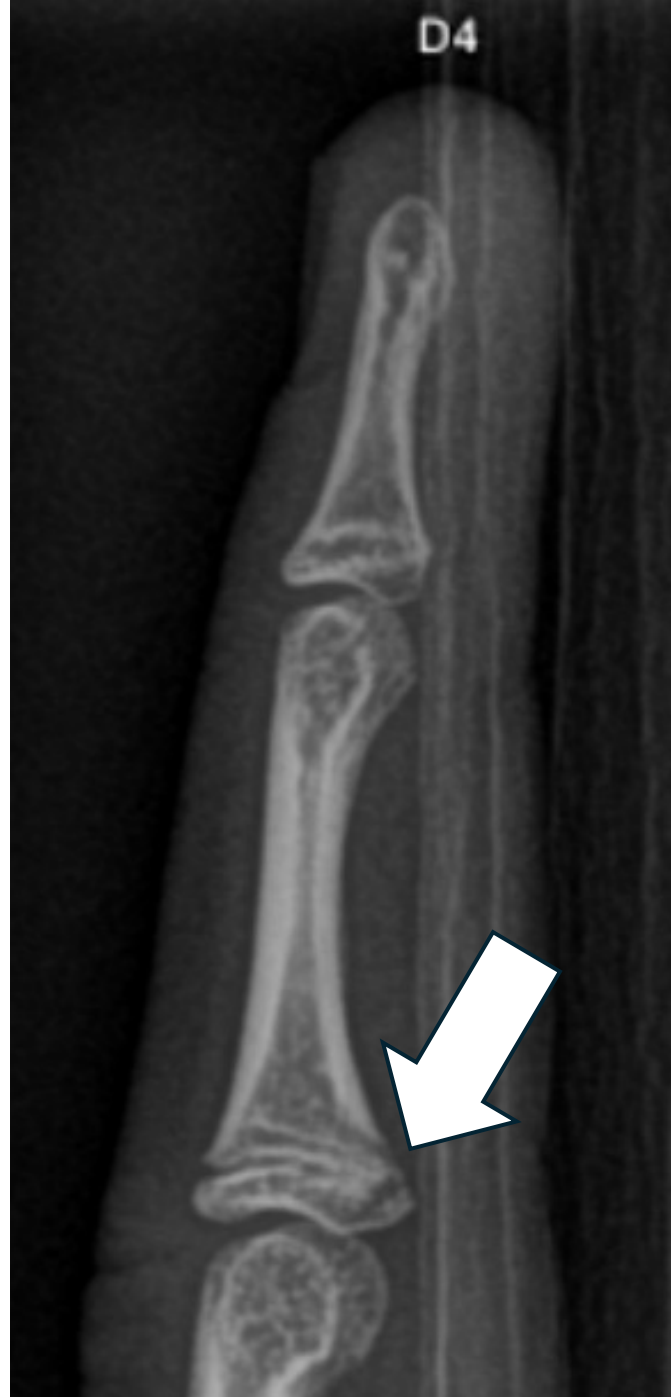

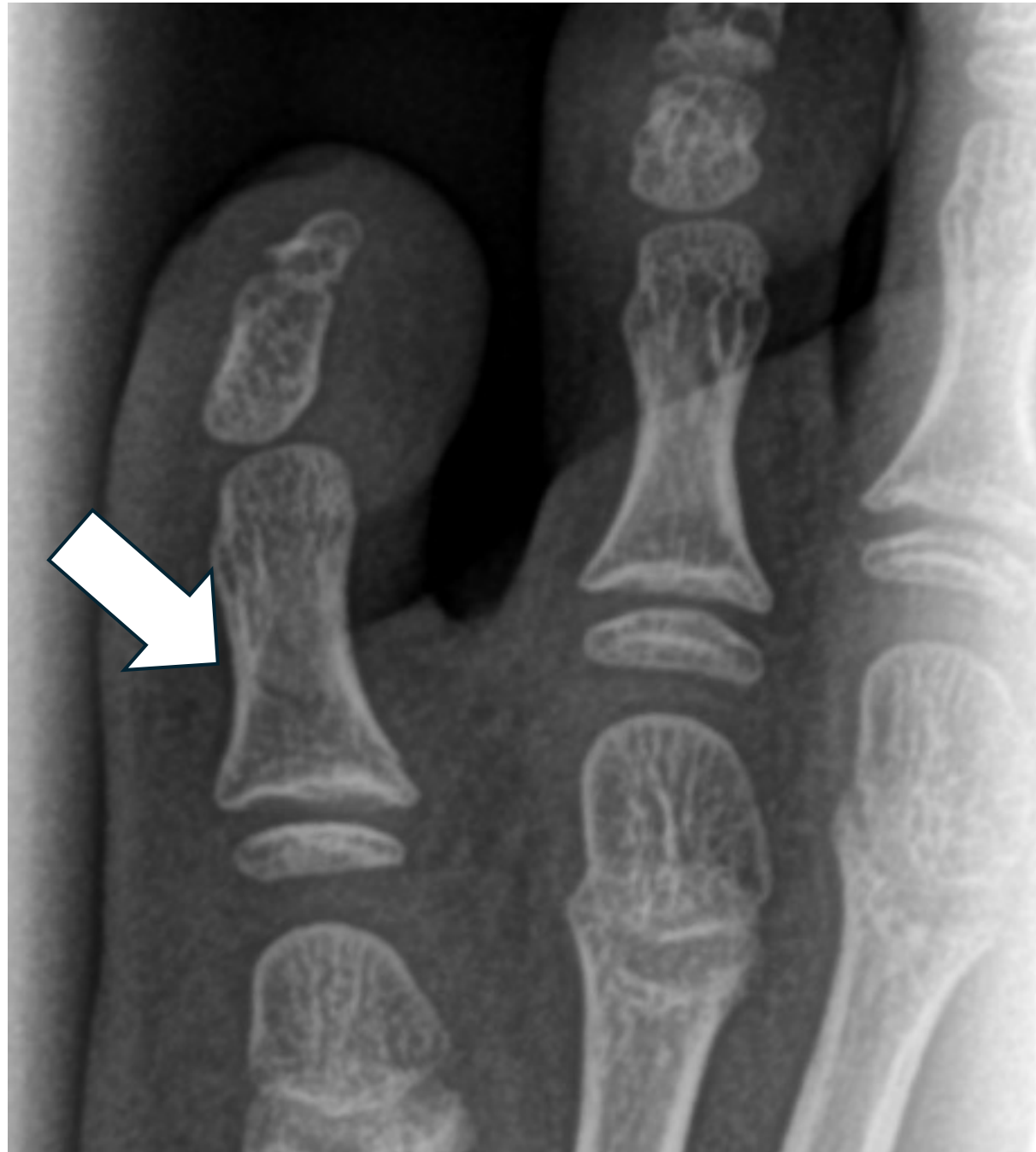

Supplement: Supplementary file 1 — ELECTRONIC SUPPLEMENTARY MATERIAL [file 330_2025_11554_MOESM1_ESM.pdf]
